# Intervention to kitchen environment for improving birth outcomes

**DOI:** 10.1016/j.lansea.2024.100446

**Published:** 2024-07-08

**Authors:** Nobuo Tsuboi, Wendy E. Hoy, John F. Bertram

**Affiliations:** a Division of Nephrology and Hypertension, Department of Internal Medicine, The Jikei University School of Medicine, Tokyo, Japan; b Centre of Chronic Disease, Faculty of Medicine, University of Queensland, Brisbane, Australia; c Department of Anatomy and Developmental Biology, Monash Biomedicine Discovery Institute, Monash University, Melbourne, Australia

We read the article by Ahmed and colleagues^1^ regarding kitchen environment and pregnancy outcomes with great interest. They conducted a cluster-randomised controlled trial for 1267 pregnant women in Bangladesh and found a 37% reduction in the incidence of low birth weight (LBW) in the intervention group with improved cookstove ventilation. They showed that increased blood carbon monoxide (CO) levels in pregnant women were associated with the increased incidence of LBW, accounting for 48.3% of the total intervention effect.

However, their article lacks mention of factors that should not be ignored when considering pregnancy outcomes. For example, pregnancy-induced hypertension (PIH) may be associated with both household CO levels^2^ and LBW.^3^ Since blood pressure measurements were reported by Ahmed and colleagues, we suggest they compare blood CO levels in the intervention and control groups based on blood pressure categories, as shown for other background factors in Table 4 of their research article.^1^

We suggest future studies investigate the following two issues regarding the association between household air pollution and the development of PIH. First, clarifying the relationship between blood CO levels and serum biomarkers of placental stress or maternal inflammatory response specific and sensitive to PIH, such as placental growth factor and soluble endothelial growth factor receptors,^3^ would be useful to further elucidate the effects of household air pollution on PIH. Second, ambulatory blood pressure monitoring has been shown to be accurate and informative in the diagnosis of PIH and is preferred over a single blood pressure measurement.^4^

## Contributors

Conceptualisation, writing–original draft: NT.

Conceptualisation, writing–review and editing: WEH and JFB.

## Declaration of interests

All the authors declare no relevant conflicts of interest.
